# Prevalence of myopic eyes in private practice and public care services in Brazil: 11 years of retrospective analysis

**DOI:** 10.5935/0004-2749.2022-0367

**Published:** 2024-09-16

**Authors:** André A. Homsi Jorge, Harley E. A. Bicas, Bruno Henrique Silva, João A. de Paula Filho, Graziela Boschetti, Marina R. de Sunti, Renata J. de Moura

**Affiliations:** 1 Hospital Oftalmológico do Interior Paulista, Araraquara, SP, Brazil; 2 Faculdade de Medicina de Ribeirão Preto, Universidade de São Paulo, Ribeirão Preto, SP, Brazil; 3 Clínica Privada, Araraquara, SP, Brazil; 4 Clínica Privada, Sorocaba, SP, Brazil; 5 Clínica Privada, Ribeirão Preto, SP, Brazil

**Keywords:** Myopia, Refractive errors, Epidemiology, Prevalence

## Abstract

**Purpose:**

This study aimed to examine the prevalence of myopic eyes over 11 years (2008-2018) in a private clinic and a public assistance service.

**Methods:**

We retrospectively evaluated 6332 individuals (12,664 eyes) between 5 and 25 years old, seen at a private clinic-CEMO (2,663 individuals) and a public service-HOIP (3,669 individuals) from 2008 to 2018. We evaluated the prevalence of myopic eyes (EE ≤-0.50) and high myopic eyes (EE ≤-6.00).

**Results:**

Sex and services did not show statistical differences. The variation in the prevalence of myopic and high myopic eyes showed a random pattern during the study period (this prevalence could not be increased). Prevalences ranged from 20.7% (in 2017) to 32.4% (in 2015) for myopic eyes and from 1.6% (in 2009 and 2016) to 3.3% (in 2015) for eyes with high myopia. The prevalence of myopia showed a statistically significant increase based on the age group.

**Conclusion:**

The prevalence of myopic eyes did not increase in our study. The mean prevalence of myopic eyes was similar in the private clinic and public service.

## INTRODUCTION

Myopia is a refractive error wherein wavefronts of the light from the infinite are refracted to a point before the retina. This may occur due to an axial cause, a refractional one, or both.

Such a refractive (axial plus refractional) error is determined by complex genetic, social, cultural, or environmental^(1^-^5)^ factors, which are not yet elucidated. The genetic factors are not related to a simple Mendelian heritage. Modern molecular technologies can identify several loci related to myopia^(3^,^5)^, and several ethnical variations and different genetic factors are responsible for its occurrence and development. Besides, the interfering social, educational, and environmental factors on its expression may also be multiple.

Recently, myopia has progressively gained attention from the scientific community. This is due to the significant increase in the prevalence of myopia in several places, mainly in the Eastern countries. Several studies have studied myopia, and theories have been advanced to explain its occurrence, as well as therapeutic measurements for its control. Myopia is the main cause of the decline in visual acuity for distant vision, affecting 1.4 × 10^9^ individuals worldwide (27% of the world population) in 2010^(6)^. In some age groups of many Asiatic countries, the prevalence of myopia is >80%. Currently, the prevalence of myopia among teenagers and young people in South Korea, Taiwan, and China is between 84% and 97%^(7^-^9)^. Evidence showed that the increased prevalence was also accompanied by increased myopia quantitative values^(8)^. Vitale et al.^(10)^ found that the prevalence of moderate myopia (between -2.00 and -7.90 D) in the USA doubled (from 11.4% in 1972-1973 to 22.4% in 1999-2000), whereas the prevalence of high myopia (>-8.00 D) increased eight times during the same interval (from 0.2% to 1.6%). The worldwide prevalence of high myopia (usually ≥-5.00 D) was 2.9% in 2010 (224 × 10^6^ individuals)^(3)^. In Brazil, studies concerning the prevalence of myopia are limited, and data were not sufficient to determine if the increasing trend affected our country. A study conducted in Goiania^(11)^ showed an increased prevalence of myopia from 9.97% (between 1995 and 2000) to 22.0% (in 2014). In 2013, another study evaluated the distribution of refractive defects in a restricted part of the Sao Paulo state and found a prevalence of myopia of 22.5%^(12)^. Considering that Brazil has 201 × 10^6^ inhabitants, we estimated that between 22 and 72 × 10^6^ individuals have myopia and between 2 and 7 × 10^6^ have degenerative myopia^(13)^.

This study aimed to observe the prevalence of myopic eyes in private practice and public assistance in two cities of the Sao Paulo state between 2008 and 2018.

## METHODS

Between 2008 and 2018, we considered only the initial ocular refraction of 6,332 subjects (12,664 eyes), including 2,663 (5,326 eyes) from a private practice *Centro Medico Oftalmológico* (CEMO) by one of the authors (A.A.H.J.) in Ribeirão Preto (SP) and 3,669 (7,338 eyes) from the *Hospital Oftalmológico do Interior Paulista* (HOIP) in Araraquara (SP), where people registered in the national public health system (SUS - *Sistema Único de Saúde*) have access. Table 1 shows the general description of the sample and information concerning the places these examinations were performed.

**Table 1:** Sample description (per eye)

Variable	CEMO (n=5326)	HOIP (n=7338)	Total (n=12,664)
**Sex, n (%):**			
Male	2308 (43.3)	3016 (41.1)	5324 (42.0)
Female	3018 (56.7)	4322 (58.9)	7340 (58.0)
**TOTAL**	5326 (100.0)	7338 (100.0)	12664 (100.0)
**Age group, n (%):**			
5-10	2406 (45.2)	2570 (35.0)	4976 (39.3)
11-15	890 (16.7)	1934 (26.4)	2824 (22.3)
16-20	836 (15.7)	1520 (20.7)	2356 (18.6)
21-25	1194 (22.4)	1314 (17.9)	2508 (19.8)
**Year, n (%):**			
2008	180 (3.4)	248 (3.4)	428 (3.4)
2009	654 (12.3)	446 (6.1)	1100 (8.7)
2010	722 (13.6)	874 (11.9)	1596 (12.6)
2011	948 (17.8)	1018 (13.9)	1966 (15.5)
2012	940 (17.6)	402 (5.5)	1342 (10.6)
2013	728 (13.7)	450 (6.1)	1178 (9.3)
2014	424 (8.0)	684 (9.3)	1108 (8.7)
2015	270 (5.1)	898 (12.2)	1168 (9.2)
2016	304 (5.7)	1108 (15.1)	1412 (11.1)
2017	120 (2.3)	550 (7.5)	670 (5.3)
2018	36 (0.7)	660 (9.0)	696 (5.5)

The inclusion criteria were age between 5 and 25 years, no associated eye disease (besides ocular refractive defects), eyes with spherical equivalents (EE) of ≤-0.50 (Group 1, myopic eyes) and ≥-0.49 D (Group 2, nonmyopic eyes) in relative (not modular) values, i.e., the signal being considered. The ocular refractive state was objectively measured using automatized refractors (Huvitz at CEMO, Canon RK-F1 at HOIP) approximately 40-60 min after cycloplegia, obtained by consecutive instillations of one eye drop each, at 1-min intervals, of solutions of proximetacaine 0.5% (for an initial ocular anesthesia), cyclopentolate hydrochloride 1.0%, and tropicamide 1.0%.

To completely analyze our results, the subjects were divided into four age groups: 5-10, 11-15, 16-20, and 21-25 years.

Special consideration was given to the group with high myopia, defined as the spherical equivalent of ≤-6.00 D.

Both, the Centro Médico Oftalmológico (CEMO) in Ribeirão Preto and the Hospital Oftalmológico do Interior Paulista (HOIP) in Araraquara, provide services for specific ophthalmological attendance, although the characteristics of their respective population differ. The CEMO is a private practice clinic extending its services to individuals with health insurance, that is, those with higher economic level (and, supposedly, educational and social), whereas the HOIP serves, exclusively, individuals depending on the public system. The locations of these centers are in cities (in a relatively prosperous Brazilian region) that are geographically close (about 90 km) and compare relatively well in several indexes of the Brazilian Institute of Geography and Statistics, IBGE)^(14)^.

The Research Ethics Committee of the University of Araraquara - UNIARA approved this study (opinion number, 4129606).

Data were analyzed using JMP SAS version 10.0 (SAS Institute Inc., Cary, NC, USA). Descriptive statistics, including frequency and percentage, were used for data presentation. Kendall tau was used to explore prevalence trends, whereas proportions were compared using the chi-square test of independence. To account for multiple comparisons, the p-value was adjusted using the false discovery rate (FDR) method. Statistical significance was established at p<0.05.

## RESULTS

**Figure 1:**
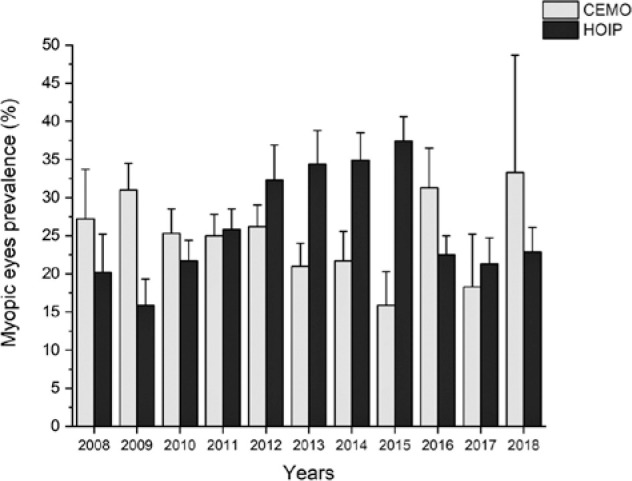
Prevalence of myopia based on the year and examination center.

Table 2 and figure 1 show the annual prevalence of myopia in each examination center.

**Table 2:** Prevalence of myopia based on the year and examination center

Year	CEMO			HOIP			p-value^*^
	Myopia (n)	Total (n)	Prevalence % (95% CI)	Myopia (n)	Total (n)	Prevalence % (95% CI)	
2008	49	180	27.2 (20.7-33.7)	50	248	20.2 (15.2-25.2)	0.1287
2009	203	654	31.0 (27.5-34.5)	71	446	15.9 (12.5-19.3)	<0.0001
2010	183	722	25.3 (22.1-28.5)	190	874	21.7 (19.0-24.4)	0.287
2011	237	948	25.0 (22.2-27.8)	263	1018	25.8 (23.1-28.5)	0.6710
2012	246	940	26.2 (23.4-29.0)	130	402	32.3 (27.7-36.9)	0.0042
2013	153	728	21.0 (18.0-24.0)	155	450	34.4 (30.0-38.8)	<0.0001
2014	92	424	21.7 (17.8-25.6)	239	684	34.9 (31.3-38.5)	<0.0001
2015	43	270	15.9 (11.5-20.3)	336	898	37.4 (34.2-40.6)	<0.0001
2016	95	304	31.3 (26.1-36.5)	249	1108	22.5 (20.0-25.0)	0.0040
2017	22	120	18.3 (11.4-25.2)	117	550	21.3 (17.9-24.7)	0.5243
2018	12	36	33.3 (17.9-48.7)	151	660	22.9 (19.7-26.1)	0.1865
**Total**	**1335**	**5326**		**1951**	**7338**	**26.6 (25.6-27.6)**	**0.0538**

* Chi-squared test (p-value adjusted by the false discovery rate [FDR] method).

The study period (2008-2018) showed no significant tendency either in the CEMO (Kendall tau=-0.16, p=0.49) or HOIP (Kendall tau=0.34, p=0.14). Moreover, if the entire study period is considered, the examinations performed showed no difference between the CEMO at 25.1% (23.9%-26.3%) and HOIP at 26.6% (25.6%-27.6%) (p=0.06).

**Figure 2:**
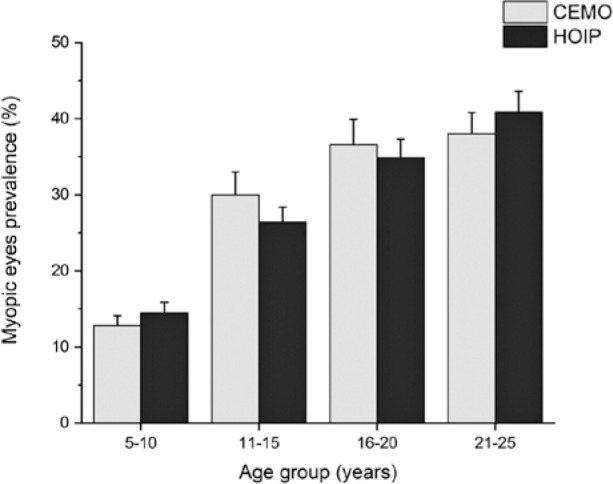
Prevalence of myopia based on the age group and service.

Table 3 and figure 2 show the prevalence of myopia in different age groups and examination centers during the study period.

**Table 3:** Prevalence of myopia based on the age group and service

Age group	CEMO			HOIP			p-value^*^
	Myopia (n)	Total (n)	Prevalence % (95% CI)	Myopia (n)	Total (n)	Prevalence % (95% CI)	
5-10	308	2406	12.8 (11.5-14.1)	373	2570	14.5 (13.1-15.9)	0.1582
11-15	267	890	30.0 (27.0-33.0)	511	1934	26.4 (24.4-28.4)	0.1582
16-20	306	836	36.6 (33.3-39.9)	530	1520	34.9 (32.5-37.3)	0.3999
21-25	454	1194	38.0 (35.2-40.8)	537	1314	40.9 (38.2-43.6)	0.1943
**Total**	**1335**	**5326**		**1951**	**7338**	**26.6 (25.6-27.6)**	**0.0538**

* Chi-squared test (p-value adjusted by the FDR method).

Each examination center showed an association between age group and prevalence of myopia (CEMO, p<0.0001; HOIP, p<0.0001).

**Figure 3:**
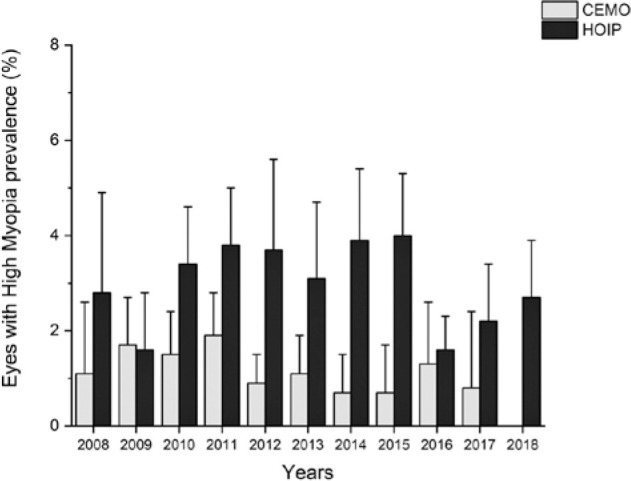
Prevalence of high myopia based on the year and examination center.

Table 4 and figure 3 show the annual prevalence of high myopia in each examination center.

**Table 4:** Prevalence of high myopia based on the year and examination center

Year	CEMO			HOIP			p-value^*^
	High myopia (n)	Total (n)	Prevalence % (95% CI)	High myopia (n)	Total (n)	Prevalence % (95% CI)	
2008	2	180	1.1 (-0.4-2.6)	7	248	2.8 (0.7-4.9)	0.3506
2009	11	654	1.7 (0.7-2.7)	7	446	1.6 (0.4-2.8)	0.8852
2010	11	722	1.5 (0.6-2.4)	30	874	3.4 (2.2-4.6)	0.0301
2011	18	948	1.9 (1.0-2.8)	39	1018	3.8 (2.6-5.0)	0.0290
2012	8	940	0.9 (0.3-1.5)	15	402	3.7 (1.8-5.6)	0.0022
2013	8	728	1.1 (0.3-1.9)	14	450	3.1 (1.5-4.7)	0.0290
2014	3	424	0.7 (-0.1-1.5)	27	684	3.9 (2.4-5.4)	0.0066
2015	2	270	0.7 (-0.3-1.7)	36	898	4.0 (2.7-5.3)	0.0290
2016	4	304	1.3 (0.0-2.6)	18	1108	1.6 (0.9-2.3)	0.7702
2017	1	120	0.8 (-0.8-2.4)	12	550	2.2 (1.0-3.4)	0.4057
2018	0	36	0.0 (0.0-0.0)	18	660	2.7 (1.5-3.9)	0.4057
Total	68	5326		223	7338	3.0 (2.6-3.4)	<0.0001

* Chi-squared test (p-value adjusted by the FDR method).

During the study period (2008-2018), the prevalence of high myopia significantly reduced in the CEMO (Kendall tau=-0.50, p=0.0344) but not in the HOIP (Kendall tau=0.04, p=0.88). Considering the entire study period, the prevalence of high myopia was significantly different between CEMO (1.3%, 1.0%-1.6%) and HOIP (3.0%, 2.6-3.4) (p<0.0001).

**Figure 4:**
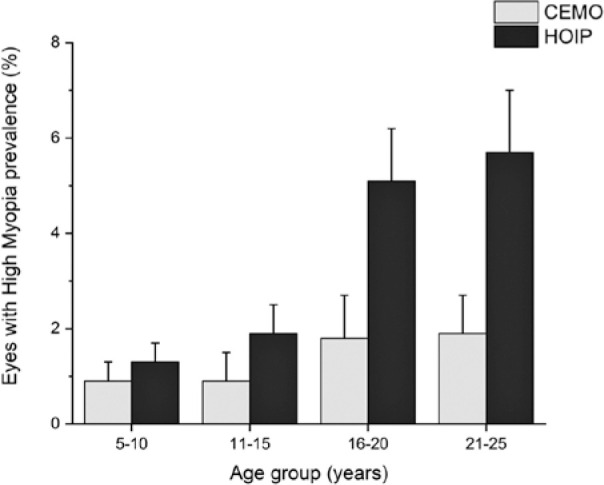
Prevalence of high myopia in the whole population based on the age group and examination center.

Table 5 and Figure 4 show the prevalence of high myopia during the study period in different age groups in the examination centers.

**Table 5:** Prevalence of high myopia in the whole population based on the age group and examination center

Year	CEMO			HOIP			p-value^*^
	High Myopia (n)	Total (n)	Prevalence % (95% CI)	High Myopia (n)	Total (n)	Prevalence % (95% CI)	
5-10	22	2406	0.9 (0.5-1.3)	34	2570	1.3 (0.9-1.7)	0.1722
11-15	8	890	0.9 (0.3-1.5)	37	1934	1.9 (1.3-2.5)	0.0607
16-20	15	836	1.8 (0.9-2.7)	77	1520	5.1 (4.0-6.2)	<0.0001
21-25	23	1194	1.9 (1.1-2.7)	75	1314	5.7 (4.4-7.0)	<0.0001
**Total**	**68**	**5326**		**223**	**7338**	**3.0 (2.6-3.4)**	**<0.0001**

* Chi-squared test (p-value adjusted by the FDR method).

An association was found between the age group and the prevalence of high myopia in both services (CEMO, p=0.0257; HOIP, p<0.0001).

## DISCUSSION

The characteristics of the two cities where the examinations were performed slightly reinforced the differences between the studied groups, that is, more socially privileged individuals were expected in the CEMO (Ribeirao Preto) than in the HOIP (Araraquara). The following shows some comparative data between the two municipalities^(14)^, obtained from the Brazilian Institute of Geography and Statistics (IBGE): population in 2022 (Ribeirão Preto: 698,259 people/Araraquara: 242,228 people), schooling in 2010 (Ribeirão Preto: 96.9%/Araraquara: 98.7%), municipal human development index in 2010 (Ribeirão Preto: 0.800/Araraquara: 0.815), infant mortality in 2020 (Ribeirão Preto: 7.12 deaths/1000 live births/Araraquara: 8.91 deaths/1000 live births), gross domestic product per capita in 2020 (Ribeirão Preto: R$ 49,476.86/Araraquara: R$ 44,813.53). Despite comparatively small differences, the data indicate better sanitary and economic characteristics in Ribeirão Preto than in Araraquara, which were the expected conditions of the individuals catered by the CEMO (richer people) than by the HOIP (poorer people).

From an initial analysis of table 1, which presents a general view of the samples, three considerations arise:

a) Although the proportions of men and women were approximately equal in the two cities, the attendance was predominantly female (58.0%), which was more than the proportion of women in the Brazilian population (51.0% in 2010)^(14)^. Other studies also reported such a trend of more women seeking ophthalmological examination^(15^,^16)^. However, the result was not related to the two different cities of examination, that is, female predominance was almost the same in either the CEMO (56.7%) or the HOIP (58.9%).

Most of the ophthalmological consults may have been motivated by refractive ocular reasons, as indicated by the forewords of two official publications of the Brazilian Council of Ophthalmology concerning ocular refraction: “The ocular refraction is the most required procedure among all others which lead a patient to an ophthalmologist”^(17)^ and “Among the multiple expected actions of an ophthalmologist, the most common is that of the optical prescriptions”^(18)^.

Notwithstanding, we can determine for those seeking ophthalmological examination whether myopia or high myopia was more frequent in women than in men. Our analysis showed that although the prevalence of myopia largely varied yearly either in women (varying between a minimum of 19.3% in 2017 to a maximum of 32.4% in 2014) or men (varying from a minimum of 20.8% in 2009 to a maximum of 33.3% in 2015), the net proportion of myopia as a possible reason for seeking ophthalmological consultation during the study period was approximately coincident in women (1909/7340=26.0%) and men (1377/5324=25.9%). No significant difference was found between the sexes.

Similar findings were also obtained with the prevalence of high myopia in men and women. During the study period, the prevalence varied in women from a minimum of 0.4% (2008) to a maximum of 3.5% (2014), and in men from a minimum of 0.3% (2012) to a maximum of 4.7% (2008). However, the net proportion of high myopia was very close in women (166/7340=2.3%) and men (125/5324=2.3%). No significant difference was found between the sexes.

b) A significant difference was found between examinations at HOIP (57.9%) and CEMO (42.1%). Such a difference is easily explained by most of the Brazilian population not having access to the most privileged type of service, such as that provided by CEMO. Besides, the number of medical doctors and doctor’s offices is greater in HOIP than in CEMO. Socioeconomic and educational levels are deciding factors for accessing medical services, such as those offered by CEMO (private consultations and health insurance). A study in 2019^(19)^ showed that only 16.1% of people with incomplete primary education had access to complementary health, that is, they were not exclusively dependent on the public system. However, this number increased to 67.6% for those with higher education. Regarding per capita household income, among those earning up to one-fourth of the minimum wage, only 3% had access to supplementary health, which increased to 88% for those earning more than fivefold of the minimum wage.

c) The examinations were more frequent in the youngest age group (39.3% between 5 and 10 years), decreased in the adolescent groups (22.3% for the 11-15-year--old group and 18.6% for the 16-20-year-old group), and slightly increased in the eldest group (19.8% for the 21-25-year-old group). This is a logical result because we expect that ophthalmological consultations were more required for the youngest age group (children) due to requirements of their respective school enrollment, the occurrence of possible visual difficulties, or simple preventive precautions taken by their parents. Incidentally, although requirements for refractive attendance progressively decreased in subjects examined at the HOIP, consultations hypothetically increased for the oldest age group at the CEMO. This may be due to this group being found more in Ribeirão Preto, where several schools of superior educational levels attract students of such age group from other neighboring cities. In addition, a 2010 study^(20)^ showed that the demand for ophthalmological care was higher in the age group between 10 and 19 years old (16.7%) compared with the age group between 0 and 9 years old (13.2%).

Table 2 and Figure 1 present the prevalence of myopia during the different years of the study. Notably, the prevalence of myopia mainly increased between 2010 and 2015, but such a tendency was reverted in the last triennial of the study (2016-2018), and the values were relative to the initial triennial (2008-2010) return. Several interesting significant differences were found between the proportion of myopia at the CEMO and HOIP, but the values float. Sometimes, the proportion was greater at the CEMO, but the proportion was significantly greater at the HOIP at other times. Myopia was observed in the CEMO and HOIP at 25.1% and 26.6% of the study population, with no significant difference between them. Therefore, the relative proportion of myopia in the examined population from 2008 to 2018 either at the CEMO or HOIP did not show a specific tendency and float between 15.9% (HOIP, 2009 and CEMO, 2015) and 37.4% (HOIP. 2015). Notably, the maximum attendance at HOIP and minimum attendance at CEMO were observed in 2015.

The study period coincided with the progressively “explosive” use of smartphones, questioning the assumption that such use should increase myopia and/or its prevalence.

Table 3 and Figure 2 show that the prevalence of myopia increases as age increases, and the difference was statistically significant between the two groups, which was consistent with other studies^(10)^. However, such an increase was approximately the same when the CEMO and HOIP were compared.

Table 4 and Figure 3 show the data for high myopia found in different years of the study. Interestingly, short “cycles” of variation of the prevalence of high myopia may be depicted over the successive years. Thus, a graph with a knurled line is suggested. The maximum and minimum prevalence of high myopia in CEMO was 1.9% (2011) and 0% (2018), respectively, whereas those in HOIP were 4.0% (2015) and 1.6% (2009 and 2016), respectively. The general proportion of high myopia at CEMO (1.3%) and HOIP (3.0%) was significantly different. This may be due to the HOIP being an acknowledged place for ophthalmological consults related to retinal affections, and the results might be biased by an increa-sed reference of eyes presenting high myopic values because since high myopia is related to vitreous and retinal detachments.

Table 5 and Figure 4 show the relationship between high myopia and age group. The proportion of high myopia was directly related to the age group. However, the progression is limited to the first three age groups because the last two presented the same results.

Table 5 and Figure 4 show the relationship between high myopia and age group to the examination center. Although the prevalence of high myopia was always greater at HOIP, a statistically significant difference was found only for the older groups (16-20 and 21-25 years). Thus, this may have occurred due to the HOIP being a regional reference for the treatment, diagnosis, and prevention of retinal problems. Similarly, the older the patient, the greater such a referential directing.

During the study period, the prevalence of myopia or high myopia did not increase. Such prevalence showed a random pattern in both services during the study period. The mean prevalence of myopia in the study period was similar in the private and public services; however, the mean prevalence of high myopia during the study period was higher in the public service.
